# Physical Stability of Chestnut Lily Beverages (CLB): Effects of Shear Homogenization on Beverage Rheological Behavior, Particle Size, and Sensory Properties

**DOI:** 10.3390/foods11203188

**Published:** 2022-10-13

**Authors:** Yao Cui, Jianxue Liu, Sihai Han, Peiyan Li, Denglin Luo, Jinying Guo

**Affiliations:** 1 College of Food and Bioengineering, Henan University of Science and Technology, Luoyang 471023, China; 2 Henan Food Raw Material Engineering Technology Research Center, Henan University of Science and Technology, Education Department of Henan Province, Luoyang 471023, China

**Keywords:** rheological properties, stability, beverages, lily bulbs, color value, high-speed shear homogenization treatment

## Abstract

The processing parameters have a crucial influence on the stability and sensory quality of beverages. The focus of this study is to observe the rheological behavior, particle size distribution, stability, color change, and sensory evaluation of chestnut lily beverages (CLB) at different rotational speeds (0~20,000 rpm) using a high-shear homogeneous disperser. The CLB system exhibited non-Newtonian shear-thinning behavior. As the homogenization speed increased (0~12,000 rpm), the viscosity increased (0.002~0.059 Pa.s). However, when the rotational speed shear continued to increase (12,000~20,000 rpm), the viscosity decreased slightly (0.035~0.027 Pa.s). Under all homogeneous conditions, the turbidity and precipitation fractions were the lowest when the rotational speed was 12,000 rpm: the sedimentation index was lowest at this point (2.87%), and the relative turbidity value of CLB was largest at this point (80.29%). The average beverage particle diameter and ascorbic acid content showed a downward trend at the homogenization speed from 0 to 20,000 rpm, whereas the total soluble solids (TSS) content followed the opposite trend. The results show that these physical properties can be correlated with different rotational speeds of homogenization. This study explained the effect of homogenization speed on CLB properties, which needs to be considered in beverage processing, where high-speed shear homogenization can serve as a promising technique.

## 1. Introduction

There is growing consumer demand for healthy, tasty, and natural functional beverages due to increasing concerns about body weight management and disease prevention [1]. Therefore, one of the focuses in the food industry is developing novel functional beverages from natural food with a potential impact on public health and the nutritional status of the population. Chestnut (*Castanea mollissima*), belonging to the *Fagaceae* plant, is widely cultivated in China [2]. vitamin C, polyphenols, calcium, magnesium, phosphorus, and potassium in chestnuts can help prevent chronic diseases such as osteoporosis, lumbar weakness, and fatigue [3]. Lily bulb (*Lilium davidii Duchartre*) is a herb of the *Liliaceae* family, which is rich in nutrients and can be used as medicine or food [4]. Lily bulbs have numerous pharmacological properties, including anti-fatigue, hypoglycemic, antidepressant, and antioxidant properties [5]. Nevertheless, owing to their high moisture content and short harvest season, the preservation of freshness is challenging and results in the limited expansion of the lily bulb industry.

To date, there are no drinks combining chestnuts with edible lilies. Combining these two raw materials to develop new composite beverages is an attractive strategy, which can not only bring more consumer attention to the nutritional value of chestnuts and edible lilies, but also reduce their losses during storage and increase the economic value. However, one of the challenges in the development of chestnut–lily beverages (CLB) is solid particle sedimentation during the sales process, because the sedimentation of solid particles has a negative impact on sensory acceptance. To solve such problems, Furkan et al. proved that using homogenization technology could improve the stability of rose nectar drinks in a previous study [6]. Moreover, Dahdouh et al. employed a high-speed shear homogenizer to improve the stability of suspended particles in orange juice [7]. Liu et al. pointed out that homogenization treatment could make the particle size of fruit and vegetable juice uniform, reduce the occurrence of stratification and precipitation, and keep the juice uniform and stable [8].

High-speed shearing homogenization (HSH) is a process of mixing, crushing, dissolving, dispersing, and homogenizing, which has been shown to not only improve the physicochemical stability of beverages, but also improve their shelf life [9]. In the high-speed homogenization process, the product is subject to the combined effects of shearing, centrifugal extrusion, impact tearing, and turbulence between the stator and rotor, creating tension that splits the droplets [10]. The homogenizing head rotating at a high speed will impact the material, and the material will become more delicate and homogenized [11]. One of the potential benefits of HSH treatment is the possibility of reducing the number of steps in beverage processing, thereby reducing nutrient loss during the processing, and saving energy and labor costs. Utomo showed that HSH was very efficient in blending miscible liquids of very different viscosities in corn and water syrup [12]. HSH treatment was also used to emulsify immiscible liquids, such as mayonnaise sauces [13]. Mueller-Fischer et al. used this technique to suspend fine air bubbles in ice cream to improve the physical properties of dairy products and jams [14].

In the field of beverage processing, the specific effects of HSH on juice properties are less studied. Hua et al. found that the acid degradation of fiber in tomato could be promoted by HSH [11]. Cardona et al. found that the stability of HSH-treated pineapple juice was affected by shearing time [15]. To our knowledge, there are no published studies on the effects of HSH on CLB. As different homogenization conditions may change some physicochemical properties of juice, the aim of the present study is to determine the effects of HSH treatment on the physical properties and sensory quality of CLB, mainly to investigate the effects of different rotational speeds on the rheological behavior, particle size, and color value of beverages. The results of this study will offer a guide to the selection of homogenization conditions and provide theoretical and technical support for the development of CLB.

## 2. Materials and Methods

### 2.1. Raw Materials and Chemicals

Chestnut kernels (5 kg) were purchased from Da Zhang Supermarket (Luoyang, China), and lily bulbs (5 kg) were purchased from Jianhuisheng Trading Co., Ltd. (Lanzhou, China). Raw materials were purchased on 23 March 2022 and stored in the refrigerator (4 °C) after purchase.

The following chemicals and distilled water were used in this experiment: ascorbic acid (>99%) was purchased from Luwei Pharmaceutical Co., Ltd. (Shandong, China); citric acid monohydrate (>99%) was sourced from Shandong Ensign Industry Co., Ltd. (Huzhou, China); xanthan gum (>99%) and CMC (>99%) were purchased from Xinjiang Meihua Co., Ltd. (Xinjiang, China); sugar was from the Lingyunhai Sugar Industry Group (Rizhao, China); and distilled water was made in our laboratory. All standards and reagents, including 2,6-Dichloroindoxyl (>98%), were obtained from Nanjing Chemical Reagent Co., Ltd., Nanjing, China.

Xanthan gum and CMC were used to improve the stability of beverage samples, citric acid and ascorbic acid were used for color protection treatment, and sugar was used for flavoring. The ratio of raw materials and additives was obtained from our previous experiments, which showed that drinks made in this formula had better sensory properties.

### 2.2. Product Development

Selected chestnut kernels and lily bulbs were mixed and cut into small pieces. The broken raw materials were heated with an electric hot plate. The pretreated raw materials (25 g of chestnut kernels and 25 g of lily bulbs) were then mixed with food additives (0.18% citric acid, 0.2% ascorbic acid, 0.06% CMC, 0.08% xanthan gum, and 7.5% sugar), and 450 mL of water was added. Then, the samples were ground for 1 min using a juicer (HR2838, Philips Home Appliances Co., Ltd., Zhuhai, China) and filtered twice with double gauze after being ground for 1 min. All the samples were stored in 500 mL sterilized glass bottles in a refrigerator (+4 °C) before being processed using a high-speed homogenizer. Samples without homogenization were used as controls.

### 2.3. HSH Treatment of CLB

Beverages were loaded into a high-shear dispersing homogenizer (AD500S-H, Wanyi Instruments Co., Ltd., Shanghai, China) immediately after removal from the refrigerator (+4 °C). The prepared CLB samples were homogenized for 10 min at various rotational speeds (4000, 8000, 12,000, 16,000, and 20,000 rpm) with the homogenizer. Before HSH treatment, the device was sterilized twice with 70% ethyl alcohol. The CLB samples were disinfected at 121 °C for 10 min using a portable pressure steam sterilizer (XFS-280HB; Xinfeng Medical Apparatus Co., Ltd., Zhejiang, China). After sterilization, the CLB (12.45 °Brix, 4.52 pH, 1.2% protein, 10.5 mg/100 mL of vitamin C) was cooled and stored at 4 °C. All CLB samples were tested microbiologically to ensure that they were safe to eat. The details and results of the microbiological tests are not the focus of this study and are, therefore, not reported here. HSH increased the outlet temperature of the samples. Therefore, after HSH treatment, the temperature of the samples was measured using a thermometer. It was demonstrated that HSH increased the temperature of juice samples (i.e., 0 rpm, 4.1 ± 0.7 °C; 4000 rpm, 18 ± 0.5 °C; 8000 rpm, 26 ± 1.1 °C; 12,000 rpm, 30 ± 1.2 °C; 16,000 rpm, 35 ± 0.4 °C; 20,000 rpm, 41 ± 1.2 °C).

### 2.4. Rheological Properties of CLB

The rheological experiments of CLB were carried out in two modes (steady state and dynamic state) using a stress rheometer (QuChen Mechanical & Electrical Technology Co., Ltd., Shanghai, China), according to the methodology of Turabi et al. The stress rheometer was fitted by parallel plate geometry (40-mm diameter) with a gap size of 1 mm [16]. The experiments were performed in isotherm conditions, which were controlled with speed and precision by a Peltier system at 25 °C and equilibrated at least 5 min before each run. First, a spoon was used to move a small amount of sample to the center of the rheometer plate. Then, the top plate was gently lowered to minimize damage to the beverage structure, and after the top plate was lowered to the proper position, excess beverage on the edge was removed and the CLB was covered with silicone oil to prevent evaporation during the measurement.

For steady-state rheological studies, the yield stress of the samples was determined by applying a shear rate sweep of 0.798–150 s^−1^ and measuring the shear stress, and the shear stress, shear rate, and apparent viscosity data were obtained directly from the instrument. The flow curves were plotted as shear stress (*τ*) versus shear rate (*γ*), and apparent shear viscosity (ηa) as a function of shear rate. The rheological behavior of different CLB samples was then fitted using a power-law model (Equation (1)) as follows:

(1) $$\tau =k\gamma^{n}$$

where *τ* is the shear stress (Pa), *k* is the consistency coefficient (Pa.s), *γ* is the shear rate (s), and *n* is the flow characteristic index. Three replicates were carried out for each determination.

For dynamic rheological studies, in order to ensure that all measurements were performed in the linear viscoelastic region, strain sweep tests (0.01 to 100% staining, frequency 1 Hz, and temperature 25 °C to determine the linear viscoelastic region) were first performed according to Zhou et al. [17]. Based on the above tests, a strain amplitude of 3% was chosen. The frequency scan provided information about the structure of the sample. Therefore, we investigated the viscoelastic behavior of CLB at 25 °C in the oscillation frequency range of 0.1~100 Hz. The frequency dependence was considered indicative of the viscoelastic properties of the material. Therefore, the variations in the energy storage modulus (*G′*) and loss modulus (*G*″) with the frequency were measured.

### 2.5. Microstructure Observation

The methodology was adapted from a previous study by Bazinet et al. [18]. To obtain micrographs of CLB particles, 3 mL beverage samples were dried for 48 h (50 °C) in a DH360BS electric thermostatic desiccator (Dejun Instrument Co., Ltd. Guangzhou, China), and the dried macromolecular solutes were pulverized into powder using a mortar and pestle. The dried sample particles were shaken on a copper platform. The particle size and distribution of the beverages were observed by feeding the CLB into a TM3030Plus scanning electron microscope (voltage: 15 kV; magnification: 1500 times) (Hitachi High-Technologies Corporation, Tokyo, Japan).

### 2.6. The Sedimentation Test and Sedimentation Index

To determine the cloudy value, a modified method of Li et al. was used [19], where beverage samples were stored upright in a 10 mL sealed glass with a screw cap at 4 °C. The samples were kept free of disturbance, other than gentle transport from the refrigerator to the lab bench for measurement. Th heights of the sediment at the bottom, the cloudy liquid in between, and a transparent liquid at the top in the sealed glass were measured using digital calipers at 0, 48, and 96 h after sample preparation, with three repeats, and the results of the sedimentation tests were determined by observing the proportion of different parts. A method based on the centrifugal sedimentation index was developed according to Xu et al. [20]. Juice samples (10 mL) were placed in graduated centrifuge tubes and centrifuged (380R, Hettich Instruments, Tuttlingen, Germany) at 4000 rpm for 15 min, after which the sediment was weighed. The sedimentation index (%, *w/w*) was expressed as the weight ratio of the centrifuged sediment to the juice sample.

### 2.7. Turbidity Measurement

Turbidity was measured according to Reiter’s method with slight modification [21]. The CLB samples and the supernatant were measured with a turbidimeter (Kuncheng Scientific Instrument Co., Ltd., Shanghai, China) after sample centrifugation (8000 rpm, 10 min). Turbidity was expressed in Turbidity Units (NTU), and relative turbidity was calculated as follows:

(2) $$T\mathrm{Relative}\;\mathrm{turbidity}\left(\%\right)=\times 100$$

where *T_1_* is the turbidity of the supernatant after centrifugation of the beverage, and *T_0_* is the turbidity of CLB.

### 2.8. Color Parameters

A colorimetric analysis was performed according to the method of Chen et al. [22] and measured using a DTQC-10A colorimeter (Oriental Instruments, Beijing, China). The supernatant was collected to measure the L* (brightness), a* (red-green), and b* (blue-yellow) values of the samples, while a white plate was used as the control. Subsequently, the color variation (ΔE∗) was calculated as follows:

(3) $$\Delta E*=\sqrt{{\left(L-L_{0}\right)}^{2\;}+{\left(a-a_{0}\right)}^{2\;}+{\left(b-b_{0}\right)}^{2}}$$

where *L_0_*, *a_0_*, and *b_0_* are the color values of the untreated beverages and L, a, and b are those of the treated samples, respectively. Differences in perceivable color can be classified as very different (ΔE > 3), different (1.5 < ΔE < 3), and slightly different (ΔE < 1.5).

### 2.9. pH and TSS

After instrument calibration with a buffer, the pH of CLB was measured using a pH meter with the probe placed in the sample by referring to the method of Gomes et al. [23].

A PAL-1 portable digital refractometer (Atago, Tokyo, Japan) was used to evaluate the total soluble solids (TSS) content [23]. The refractometer was calibrated to zero with distilled water and a drop of the beverage was deposited on the refractometer prism, from which the results could be observed. The TSS content of the beverage was recorded as °Brix, and the results were presented as the mean ± standard deviation at room temperature (25 °C).

### 2.10. Ascorbic Acid

Ascorbic acid was analyzed according to the procedure described by Ti et al. [24]. The samples (5 mL) were diluted to 100 mL with distilled water at 4 °C, mixed with 2 mL of extract and 25 mL of 20% glacial acetic acid, and then shaken and titrated with sodium 2,6-dichloroindoxyphene solution until it was completely pink and did not fade within 15 s. A standard curve was drawn using ascorbic acid standards, and the results were expressed in mg/mL.

### 2.11. Organoleptic Evaluation

This study was approved by the Ethics Committee of Henan University of Science and Technology. The participants were asked to sign an informed consent form prior to participation in the study. Beverage samples (25 mL) were stored at 4 °C for 24 h and sensory testing was performed by 20 non-trained consumers. The overall acceptability, aroma, appearance, mouthfeel, and taste of the CLB were separately evaluated by each participant using a 9-point structured hedonic scale (1 = dislike extremely, 2 = dislike very much, 3 = dislike moderately, 4 = dislike slightly, 5 = neither like nor dislike, 6 = like slightly, 7 = like moderately, 8 = like very much, and 9 = like extremely). The overall acceptability refers to the overall preference score of CLB expressed by participants, the smell refers to the volatile smell of CLB inhaled by the participants through the nose, and the appearance refers to the score of the color and uniformity of the beverage measured by the eyes of the participants. Mouthfeel refers to participants’ ratings of its viscosity, and taste refers to participants’ ratings of whether it tastes harmonious. The panelists were instructed to drink the samples and rinse their mouths with water at room temperature between sample evaluations. A sensory analysis was performed with minimal interference in the laboratory, which was free from noise or off-odor.

### 2.12. Statistical Analysis

Data were collected using Statistica 7.0 (StatSoft Inc., Tulsa, OK, USA) and plotted using Origin 12.0 (OriginLab Inc., Northampton, MA, USA). Data were analyzed by a one-way analysis of variance (ANOVA) followed by Duncan’s multiple comparison using SPSS 16.0 software (IBM Inc., Armonk, NY, USA). The level of significance was set at *p* < 0.05. All experiments were repeated in triplicate.

## 3. Results and Discussion

### 3.1. Flow Behavior and Shear Viscosity of Beverages

#### 3.1.1. Steady-State Rheological Properties

**Figure 1:**
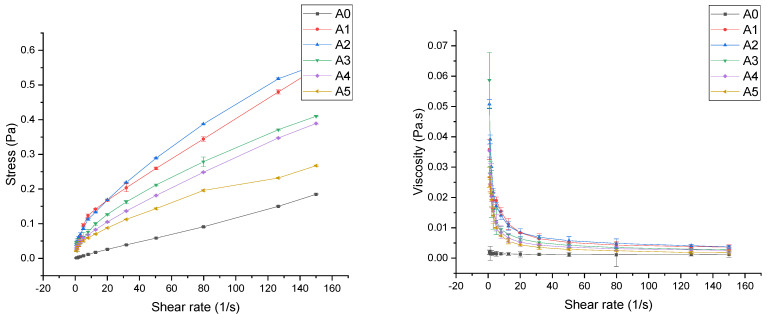
Steady-state rheological curves of CLB at different rotating speeds. Three independent measurements were made on each sample and the results are presented as the mean with error bars representing the standard deviation. In the figure, A0: not homogenized; A1: 4000 rpm; A2: 8000 rpm; A3: 12,000 rpm; A4: 16,000 rpm; A5: 20,000 rpm.

Rheological qualities are utilized in workflow system design, product formulation, and beverage manufacturing processes [25]. The shear effects on the processing of CLB samples at different rotational speeds are described by the response of shear stress-shear rate and viscosity-shear stress, which can help us explore the relationship between the homogenization conditions and physical properties of beverage systems. The flow curves of CLB under different homogeneous conditions are shown in Figure 1. All the specimens exhibited non-Newtonian characteristics and shear-thinning (pseudoplastic) behavior, and the apparent viscosity (*η*, Pa.s) decreased and shear stress increased with increasing the shear rate (*γ*, s^−1^). HSH treatment did not alter the flow pattern of CLB. Pseudoplastic behavior was also observed in the rheological analysis of grape whey juice by Amaral et al. [26]. There was a change in the apparent viscosity of CLB after HSH over the entire shear rate range, suggesting that HSH slightly affected the rheological properties of CLB. The difference in rheological properties after CLB treatment may be related to the fragmentation of aggregated macromolecules such as starch and pectin [27]. A non-Newtonian fluid is a fluid whose shear stress and shear strain rate are not linearly related [28,29]. Fangary et al. found that different rheological behaviors were related to the alignment of the microstructure, flow direction, and the disintegration of agglomerated particles [30].

At a shear rate of 0.798 s^−1^, the shear viscosity (0.002~0.059 Pa.s) and yield stress (0.001~0.047 Pa) increased by rotations ranging from 0 to 12,000 rpm. This was probably because the acceleration generated by homogenization resulted in strong shear and fragmentation forces in the beverage system, and this tendency intensified with increasing the rotational speed. Changes in the average molecular weight and chain length may affect the frequency of beverage particle collisions, resulting in shear viscosity changes of the beverage system [31]. The pectin molecule is one of the main factors of physical interaction, and the viscosity of the juice is also affected by the properties of the pectin. With the change in the homogenization conditions, its apparent viscosity, average molecular weight, and particle size will change [32]. The viscosity (0.059~0.02 Pa.s) and shear stress (0.047~0.021 Pa) of CLB tended to decrease at rotational speeds of 12,000~20,000 rpm. Bi et al., studying the properties of soy protein isolate (SPI) and kappa-carrageenan (kappa-KARA) mixed dispersions, found that high-speed shear treatment increased the stability of the mixing system, but the apparent viscosity decreased when the rotating speed was higher than 8000 rpm, which may be because the destruction effect gradually became stronger than the mixing effect [33]. Yield stress is a critical quality control parameter in industrial processing, transportation, or storage. The yield stress (*τ_0_*) values of CLB under different homogenization conditions were different, indicating that the apparent viscosity had a trend of change. Variations in shear stress values may be related to differences in the particle volume fraction, interparticle forces, and particle size [34]. Since viscosity is related to intermolecular interactions, the increase in the rotational speed will change the intermolecular energy in the beverage system, resulting in changes in the molecular binding of the beverage, and further changes in viscosity. According to Christiaens et al., mechanical treatment during HSH can lead to the fragmentation and destruction of plant cells in beverages, which may also lead to changes in viscosity [35]. Dahdouh et al. found that when the original compositions of some food and dietary fiber or other additives were changed in food formulations, as well as the technological processes applied, some physical properties, such as rheology and particle size, would be affected [7]. Bi et al. said that the change in the particle size would alter the average distance between particles and the interaction of particles, which would lead to a change in viscosity [27].

#### 3.1.2. Dynamic Frequency Scan

**Figure 2:**
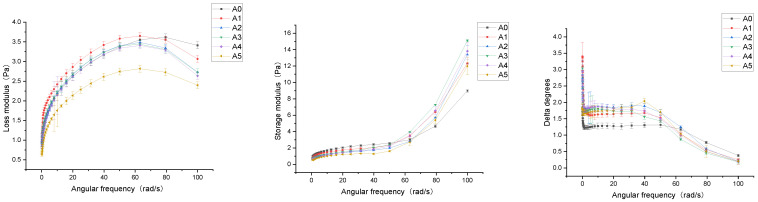
Variations in storage modulus (*G′*), loss modulus (*G″*), and delta degrees of CLB in a dynamic frequency scan. Three independent measurements were made on each sample and results were presented as the mean with error bars representing the standard deviation. In the figure, A0: not homogenized; A1: 4000 rpm; A2: 8000 rpm; A3: 12,000 rpm; A4: 16,000 rpm; A5: 20,000 rpm.

To assess whether the beverage system was strongly flocculated, we used dynamic frequency experiments to estimate the resistance of the beverage to deformation [36]. correlated with the solid nature of the beverage, and the loss modulus (*G″*) reflects liquid-like properties. *G″/G′* is the delta representing the flocculation power of the beverage system (Figure 2) [26]. In the current study, when the fixed strain was 3%, the storage modulus (*G′*) and loss modulus (*G″*) of all samples demonstrated a relatively strong frequency dependence, accompanied by a delta reduction with increasing frequency, indicating that the rheological properties of the beverage were significantly influenced by the deformation rate at frequencies from 0.1 to 100 rad/s. The *G′* and *G″* of the beverage samples increased with the increase in frequency, which showed weak gel dynamic rheological properties, and Ptaszek et al. said that the increase in frequency would destroy the chemical bonds between molecules [37], which may be related to the changes of G″ and *G′*. Variations in *G′* and *G″* in CLB may be due to changes in the particle morphology of beverages and different homogenization treatments affecting particle-to-particle interactions [38,39]. Augusto et al. found a similar phenomenon when evaluating the effect of homogenization on the rheological behavior of tomato juice, and their study claimed that the observed difference in the rheological behavior in terms of viscoelasticity was due to the breakdown of suspended particles during processing [40]. Furthermore, at the same frequency, the *G″/G′* of CLB decreased with increasing the shear rate, which might be because a high shear rate heated the beverage, causing the force between the protein, starch, and other biological macromolecules in CLB to decrease with the increase in temperature and hindering the formation of the intermolecular network structure [41]. The differences in the flow properties of CLB after HSH treatment could be partly due to the solubilization of large particles such as starch and pectin in beverages [42]. Among other researchers, Bi et al. found that the HSH treatment of soy protein isolates caused the hybrid system to become stronger and more solid by observing rheological behavior [43], and HSH treatment also helped stabilizers disperse into the system more uniformly. Dahdouh et al. reported that in the effect of high-speed shear on the rheological behavior of orange juice, different shear conditions simultaneously induced significant changes in the particle size distribution and rheological properties of the juice (potential increase in cohesive energy between particles) [7]. Our results indicate that resistance to the deformation of the beverage system was related to different homogenization conditions.

### 3.2. Particle Size Analysis

**Figure 3:**
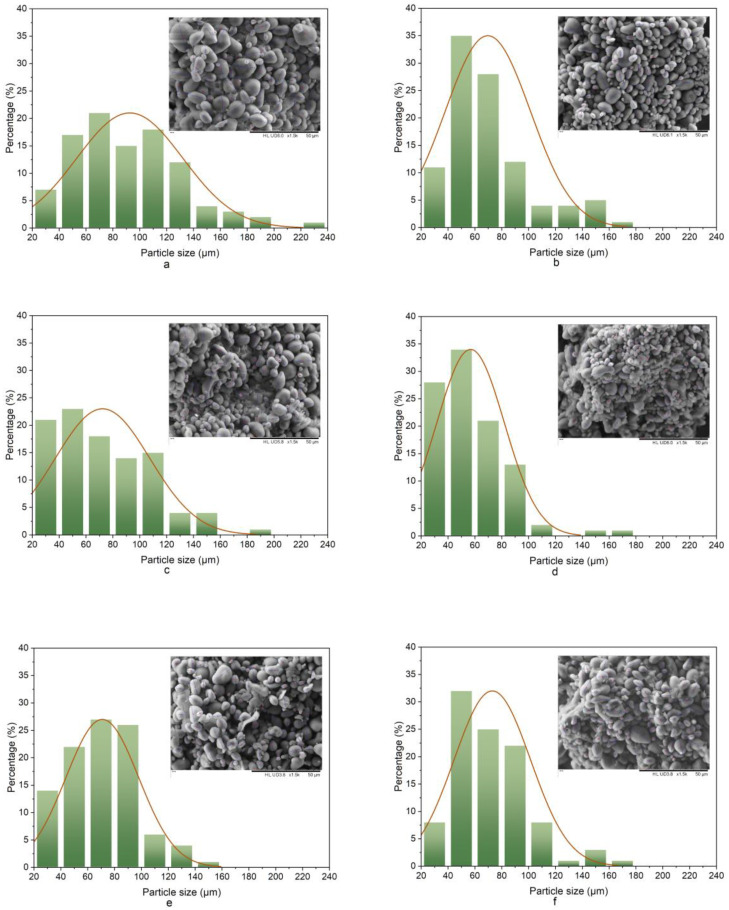
SEM image and particle size analysis of solid particles (1500×). (**a**): not homogenized; (**b**): 4000 rpm, 10 min; (**c**): 8000 rpm, 10 min; (**d**): 12,000 rpm, 10 min; (**e**): 16,000 rpm, 10 min; (**f**): 20,000 rpm, 10 min.

The size distribution of the beverage microparticles was determined by scanning electron microscopy. The effect of HSH on the particle size of CLB is shown in Figure 3. Non-homogenized CLB contained a wider range of particle sizes (27.4~228.75 µm), often with the aggregation of particles or incorporation of large droplets. As expected, the average particle size of the beverage decreased with increasing the rotational speed compared with that of non-homogenized control samples. The particle sizes ranged from 24.87 to 170.83 μm, with the majority between 20 and 560 μm after the sample was homogenized at 20,000 rpm. The mean particle size of 56.77 ± 0.02 μm was 38% smaller than that of the heterogeneous samples, which indicated that larger particles were broken into smaller ones. A previous study identified that the rheological behavior of acidic milk beverages was related to their particle diameter and spatial distribution, which affected their stabilization [44]. Kieserling et al. reported that high-speed shear homogenization caused the disintegration and fracture of crude fibers in fruit as a key mechanism for changes in the particle diameter [31]. When the diameter of beverage microparticles changed, the interactions between the dispersions became more unstable, which might also change the rheological behavior of CLB [40]. Zhou et al. also reported that a change in the particle size was associated with the homogenization speed [45].

### 3.3. The Sedimentation Test and Settlement Index

**Figure 4:**
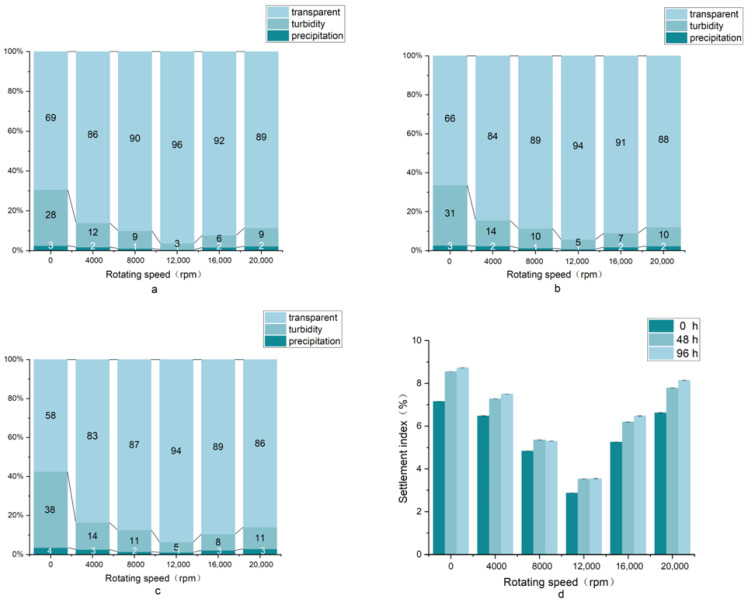
Proportion (%) of turbidity, transparency, and precipitation in CLB samples. (**a**): 0 h; (**b**): 48 h; (**c**): 96 h; (**d**): sedimentation index.

In pursuit of a straightforward, rapid, and objective evaluation of the beverage system, the intuitively visible sediment volume ratio in juice could be estimated using a sedimentation test [46]. The aggregation behavior of juice microparticles is controlled by two mechanisms: flocculation and settling. Flocculation is a phenomenon in which particles combine to form aggregates, increasing the effective particle size. Settling refers to the sedimentation of flocculated particles from the suspension to the bottom of the vessel. Homogenization may change them into cloudy matter. The turbidity and precipitation of the beverage were high in the non-homogenization setting, and sedimentation was reduced at 4000, 8000, and 12,000 rpm. When the rotational speed exceeded 12,000 rpm, a higher turbidity and higher precipitation fraction were formed in CLB (Figure 4). Beverages contained trace components such as pectin, proteins, lipids, cellulose, hemicellulose, etc., and differences in the interactions between these components might have an effect on the aggregation of particles in the beverage [47]. As the rotating speed became large enough to form smaller beverage microparticles, cloud stability underwent a rapid drop owing to the weakening of interactions between flocculation networks, which led to an increase in the turbidity and precipitation [48]. HSH treatment reduced the turbidity and sedimentation fractions of CLB compared to non-homogenized samples. These results are similar to those of Bi et al., who also found that HSH reduced the “phase separation” function of the system to a certain extent [43].

The settlement index of CLB recorded at a speed of 0~20,000 rpm is shown in Figure 4d. These data indicated that HSH could reduce the sedimentation index. The subsidence index gradually decreased with an increase in the rotational speed. This may be because larger particles aggregated faster than smaller particles. CLB could acclimate to 12,000 rpm and had the lowest subsidence index (2.87%) by this time. However, the subsidence index did not decrease when the speed exceeded this intensity. In high-shear homogenization, an excessive rotating speed was not conducive to the physical stability of the beverage, although homogeneous strength played an essential role in its quality. A possible explanation was as follows: increasing the speed caused the number of beverage microparticles to increase, therefore, the existing stabilizer concentration was not sufficient to cover them, and an additional stabilizer is required for the increased particle area [46]. This result suggested that the sedimentation index of CLB at 0, 48, and 96 h gradually increased as the storage time increased. A possible explanation for these results could be that the overnight buildup of organic acids in the cells of the beverage during storage decreased the tissue pH, and the molecular structure of the stabilizers used to stabilize the beverage system was disturbed during this acidification process, resulting in a decrease in physical stability [49].

### 3.4. Turbidity

The relative turbidity value reflects the stability of CLB, with a higher value indicating better stability. As can be seen in Table 1, the relative turbidity value of the beverage increased from 0 to 12,000 rpm and reached the maximum (80.29 ± 0.9%) at 12,000 rpm. When the rotational speed exceeded 12,000 rpm, it showed a downward trend. This might be related to the increase in beverage temperature caused by an excessive rotation speed, and the temperature increase would cause the coagulation of thermosetting protein in the juice, which would lead to the sedimentation of particles in the juice and reduce the stability of the beverage system [21]. Overall, the relative turbidity value of unhomogenized CLB was the lowest (56.24 ± 2.24%) among all samples, which indicated that homogenization helped improve the physical stability of CLB. Suspension particles in beverages contained proteins, lipids, pectin, hemicellulose, cellulose, and other minor constituents in a complex mixture [50]. Particle size and rheological properties in beverages could affect the turbidity of the beverage system [26]. Kristiani et al. stated that changes in the particle size or viscosity could affect the turbidity of apple juice [51].

### 3.5. Color Value

As can be observed, the rotating speed of 20,000 rpm significantly (*p* < 0.05) affected the color properties of CLB (Table 1). The value of L* at 20,000 rpm decreased significantly (*p* < 0.05) with increasing the rotation speed, but not from 0 to 16,000 rpm. However, there was no significant difference in the a* and b* values between CLB samples treated at different speeds. A decrease in the L* value showed a decrease in brightness, which was directly related to juice browning [52]. The cloud of juice might also have an effect on the color value, as the reflected light was affected by the cloud [53]. Chestnuts and lilies in CLB with higher contents of ascorbic acid and phenolics were prone to browning during processing and storage. Wang et al. said in the study of strawberry juice that the reduced lightness in samples was possibly attributed to oxidative browning [54]. The difference in the L value may be due to the degradation of vitamin C, resulting in the mild browning of the juice [55]. That the beverage had an increased occurrence of browning reaction could, therefore, be attributed to the increase in the temperature of the beverage system under high-speed homogenization treatment [56]. Suarez-Jacobo et al. found that an elevated temperature caused the discoloration and browning of apple juice, which involved several reactions, including the Maillard reaction and pigment destruction [57]. We could not rule out that different rotation speeds, apart from the effect on the suspended solid granules of beverages, could have an effect on the visible spectrum of the beverage system, which would cause a change in the color value [54]. Additional uncertainty arose in that the increasing L* value with a rotational speed could be attributed to the decreasing particle size, which increased the particle surface area and introduced more oxygen into the beverage, resulting in a change in color [17]. Excessive rotational speed caused severe mechanical damage that might seriously impair cells and cell membranes, which might also affect color values [58].

### 3.6. pH and TSS

The pH value is essential for assessing the beverage quality. Compared to the control group, no changes in the pH value of the beverage were observed at 4000~16,000 rpm, whereas a slight decrease was found at 20,000 rpm (Table 1). The reduction in the pH value may partly be explained by the increase in homogenization speed, which causes the hydrolysis of starch, an increase in acidity, and the accumulation of organic acids [59]. Reiter et al. claimed that acidification caused protein precipitation and cloud particle aggregation in carrot juice. The aggregation behavior of granules resulted in decreased juice stability [21].

Rotation speeds from 0 to 12,000 rpm did not cause significant changes in the TSS content. When the rotation speed exceeded 12,000 rpm, the TSS content began to increase and reached the maximum at a rotation speed of 20,000 rpm. Plant cell walls and membranes are cleaved during high-speed shearing, which may be the main reason for the increase in soluble solids in CLB [8]. Changes in TSS may affect the rheological behavior and particle size, resulting in changes in particle interactions, which impact the beverage stability [60]. Under more severe homogenization conditions, cell wall damage in plant-based beverages intensifies, and the release of natural ingredients, e.g., cell wall polysaccharides, dramatically increases in CLB. Changes in the properties of these ingredients may lead to beverage structure changes, affecting beverage stability [61].

### 3.7. Ascorbic Acid

High temperature and oxidation are important factors in the degradation of ascorbic acid. As shown in Table 1, it is apparent that the ascorbic acid content under unprocessed conditions was almost comparable to that at 4000 and 8000 rpm, but began to decrease significantly after more than 8000 rpm. When the sample was homogenized at 20,000 rpm, ascorbic acid was significantly reduced (42.6%), compared with that of the unhomogenized samples. This phenomenon may relate to the fact that the increased rotating speed in homogenization leads to a temperature rise and increased damage to plant cell walls and organelles, which may affect the oxidative decomposition of ascorbic acid in fruit [62]. Zcw et al. found that a temperature increase would destroy the structure of ascorbic acid, cause thermal decomposition, and accelerate the oxidative degradation of the acid to a certain extent. Further, some of the ascorbic acid might also undergo anaerobic degradation, resulting in a decrease in its content [63].

### 3.8. Sensory Assessment

Sensory parameters could be affected by product processing, as well as the composition and microstructure. Appearance scoring is an important method for beverage assessment. The average appearance score was improved in the homogenized group compared with that in the control group. A possible explanation could be the poor stability of the unhomogenized CLB layer and the precipitation in the beverage. The appearance scores of CLB followed similar outcomes at different speeds. However, these differences were not statistically significant. Aroma is an essential component of the CLB quality as it can contribute to the sensory attributes of pleasant or unpleasant beverages. In the sensory evaluation, the aroma score of homogenized CLB strongly decreased with an increase in the rotational speed. It is possible that these results were due to the volatile off-odors of beverages treated with high-speed shearing, which caused more severe losses. Mouthfeel is the physical sensation associated with the presence of beverages in the mouth. Compared with the control group, the mouthfeel of the beverage was improved when CLB was treated at 4000 rpm and 8000 rpm, but the score showed a downward trend when it exceeded 8000 rpm. Except for the samples treated at 20,000 rpm, the sensory scores of other CLBs were greater than 6.0. Our findings showed that an excessive rotational speed is not conducive to the improvement of the CLB sensory score. According to these data, we can infer that most panelists were satisfied with the CLB sensation. However, given the limited number of participants, these results should be interpreted with caution.

## 4. Conclusions

When processing juice with HSH, it is important to consider the effects of high shear rates on the physical stability and sensory perception of the beverage. In this study, the effects of different levels of homogenization (4000~20,000 rpm) on physical properties such as the rheological properties, microstructure, and sedimentation index of CLB were investigated. Maximum shear viscosity (0.059 Pa.s) and yield stress (0.047 Pa) were obtained at a rotational speed of 12,000 rpm. Particle size reduction after HSH treatment was observed by optical microscope, and the average particle size decreased by 38% at 20,000 rpm. At this point, the brightness value decreased. This contributes to our understanding of the effects of different homogenization conditions on the physical properties of CLB. A number of limitations in our study should be noted, including the fact that we only studied the effects of different rotational speeds on CLB physical properties and surface characteristics. We believe that the conclusions drawn in this work will contribute to the production of high-quality CLB and help ensure the physical stability of the botanical complex beverage before complex chemical analysis. In the future, it is necessary to further investigate the bioactive substances, enzymatic activities, and storage stability of CLB under different homogeneous conditions: Cui, Y.; Liu, J.; Han, S.; Li, P.; Luo, D.; Guo, J.

## Figures and Tables

**Table 1:** Other physicochemical characteristics and physical properties of homogenized beverages.

Rpm	0	4000	8000	12,000	16,000	20,000
L	81.0 ± 0.5 ^a^	81.2 ± 0.1 ^a^	80.5 ± 0.1 ^a^	80.9 ± 0.2 ^a^	80.7 ± 0.4 ^a^	74.3 ± 0.2 ^b^
a	−3.3 ± 0.3 ^a^	−3.3 ± 0.1 ^a^	−3.3 ± 0.1 ^a^	−3.3 ± 0.1 ^a^	−3.3 ± 0.1 ^a^	−2.8 ± 0.1 ^a^
b	6.8 ± 0.1 ^a^	7.0 ± 0.1 ^a^	6.6 ± 0.1 ^b^	6.7 ± 0.1 ^a^	6.7 ± 0.1 ^a^	6.7 ± 0.0 ^a^
ΔE	0	0.63	0.63	0.31	0.38	0.68
pH	4.5 ± 0.2 ^a^	4.57 ± 0.14 ^a^	4.4 ± 0.1 ^a^	4.3 ± 0.1 ^a^	4.22 ± 0.1 ^a^	3.81 ± 0.1 ^b^
TSS	12.4 ± 0.2 ^b^	12.9 ± 0.2 ^b^	13.5 ± 0.1 ^b^	13.7 ± 0.1 ^b^	14.1 ± 0.1 ^a^	14.4 ± 0.2 ^a^
Ascorbic acid	10.5 ± 0.1 ^a^	9.64 ± 0.2 ^a^	7.8 ± 0.1 ^b^	7.85 ± 0.2 ^b^	7.4 ± 0.1 ^b^	6.0 ± 0.1 ^c^
Appearance	5.2 ± 1.0 ^b^	5.7 ± 0.8 ^ab^	5.9 ± 1.3 ^a^	6.6 ± 0.9 ^a^	5.6 ± 1.1 ^ab^	6.1 ± 1.2 ^a^
Aroma	6.2 ± 1.0 ^a^	6.2 ± 0.8 ^a^	5.2 ± 0.5 ^b^	6.2 ± 0.7 ^a^	4.7 ± 1.6 ^b^	4.5 ± 0.9 ^b^
Mouthfeel	5.2 ± 1.1 ^b^	6.3 ± 1.4 ^a^	6.1 ± 1.2 ^ab^	6.8 ± 0.8 ^a^	7.1 ± 0.6 ^a^	5.1 ± 0.9 ^a^
Taste	5.4 ± 1.8 ^b^	6.0 ± 1.9 ^a^	6.7 ± 1.3 ^a^	6.5 ± 1.1 ^a^	6.2 ± 1.2 ^a^	5.2 ± 1.2 ^b^
Overall acceptability	5.9 ± 1.0 ^a^	6.1 ± 1.4 ^a^	6.5 ± 1.2 ^a^	6.3 ± 1.2 ^a^	7.1 ± 0.7 ^a^	5.5 ± 1.2 ^b^
Temperature	4.1 ± 0.7 °C	18.1 ± 0.5 °C	26.6 ± 1.1 °C	30.5 ± 1.2 °C	35.3 ± 0.4 °C	41.0 ± 1.2 °C
Relative turbidity	56.2 ± 2.2 ^c^	70.2 ± 1.3 ^b^	72.1 ± 1.2 ^b^	80.3 ± 0.9 ^a^	76.4 ± 2.1 ^ab^	72.1 ± 2.6 ^b^

TSS: Total soluble solids (°Brix). Ascorbic acid content: (mg/100 mL). Three independent measurements were performed for each sample, and the results are indicated as the mean ± standard deviation. Different letters in the same row indicate significant differences (*p* < 0.05) between the means.

## Data Availability

The data presented in this study are available on request from the corresponding author.
